# The TAKE-IT study: aims, design, and methods

**DOI:** 10.1186/1471-2369-15-139

**Published:** 2014-08-30

**Authors:** Bethany J Foster, Ahna Pai, Huaqing Zhao, Susan Furth

**Affiliations:** 1 Montreal Children’s Hospital, 2300 Tupper St, E-222, Montreal, Quebec H3H 1P3, Canada; 2 Department of Pediatrics, McGill University, Montreal, Quebec, Canada; 3 Department of Epidemiology, Biostatistics, and Occupational Health, McGill University, Montreal, Quebec, Canada; 4 Department of Pediatrics, Cincinnati Children’s Hospital Medical Center, 240 Albert Sabin Way, MLC 7039 S.9552, Cincinnati, OH 45229, USA; 5 Department of Clinical Sciences, Temple University School of Medicine, MERB 955, 3500 N. Broad St., Philadelphia, PA 19140, USA; 6 Department of Pediatrics, Children’s Hospital of Philadelphia, 3401 Civic Center Boulevard, Ste 1E15, Philadelphia, PA 19104, USA; 7 Center for Clinical Epidemiology and Biostatistics, University of Pennsylvania, Philadelphia, PA, USA

**Keywords:** Adherence, Randomized trial, Adolescent, Intervention, Kidney transplantation

## Abstract

**Background:**

Effective interventions to improve immunosuppressive medication adherence among adolescent and young adult kidney transplant recipients are desperately needed. This paper describes the aims, design, and methods of the Teen Adherence in Kidney transplant, Effectiveness of Intervention Trial (TAKE-IT) study.

**Design and methods:**

TAKE-IT is a multicentre, prospective, open-label, parallel arm randomized controlled trial that aims to determine the effectiveness of a clinic-based intervention, including *educational, organizational,* and *behavioural* components, in improving immunosuppressive medication adherence among adolescent and young adult kidney transplant recipients. Individuals between 11 and 24 years of age who are at least 3 months post-transplant and followed in one of the eight participating pediatric kidney transplant programs, or their affiliated adult transplant programs are eligible to participate. All participating centers are tertiary care pediatric hospitals in Canada or the United States. Adherence is monitored using an electronic multi-dose pillbox for all participants during a 3-month run-in period, followed by a 12-month intervention interval. The primary outcome is ‘taking adherence’, defined as the proportion of prescribed doses of immunosuppressive medications that were taken, as measured using electronic monitoring.

All participants meet with the study ‘Coach’ at 3 month intervals. The intervention, administered by trained lay personnel, targets common adherence barriers. In addition to forming an Adherence Support Team, intervention participants identify personal barriers to adherence and use Action-focused problem-solving to address them, have their electronic adherence data fed back to them, and have the option to receive email, text message, or visual cue dose reminders. Participants in the control group meet with the coach but do not receive the other components of the intervention. The study aims to have 75 participants in each group complete the study.

**Discussion:**

Since recruitment began in Feb. 2012, 198 adolescents have been approached to participate, of whom 130 have completed a baseline visit. As of March 31, 2014, 125 had been randomized, and 86, 68, 61, and 50 participants had completed 6-month, 9-month, 12-month, and 15-month visits respectively.

**Trial registration:**

Clinicaltrials.gov registration NCT01356277 (May 17, 2011).

## Background

Adolescence and emerging adulthood [1] is a high-risk period for kidney transplant recipients. Graft failure rates begin to rise at about 11 years of age, peak in the interval between 17 and 24 years, and decrease thereafter [2]. Non-adherence to immunosuppressive therapy may be the most important factor contributing to poor graft survival in this age group [3-5]. In a study of pediatric kidney transplant recipients, each 10% decrement in adherence (estimated using the medication possession ratio determined from claims data) was associated with an 8% higher hazard of graft failure [6]. Although numerous risk factors for non-adherence have been identified [7,8], to date no interventions have been developed that systematically target these risk factors in the pediatric kidney transplant population.

There are numerous reasons for poor medication adherence. Most non-adherence is ‘unintentional’ [9], and believed to be related to inadequate organizational skills and/or problem-solving abilities, or to complexity of the medical regimen. Forgetting was the most commonly stated reason for missing medications (56%) in one study of adolescent renal transplant recipients [10], and the second most common (29%), after organizational problems (58%), in another study [11]. Other modifiable risk factors for non-adherence in adolescents with transplants include lack of parental supervision, poor parent-patient communication, poor medication and disease knowledge, lack of pillbox, and complex medication regimens [3,4,6-8,10,11].

An effective adherence intervention must address the most common, and most powerful, determinants of non-adherence. Prior studies of mainly adult transplant recipients [12] and children and adolescents with other chronic illnesses [13-15] indicate that effective interventions include education in conjunction with some combination of adherence monitoring, promotion of problem-solving, goal-setting, development of routines, and/or adherence support. Adherence support from a key person from outside the healthcare team – called a "personal trainer" [15] – and text message dose reminders also show promise in improving adherence [16]. A randomized trial in 150 adult kidney transplant recipients found significantly better adherence and lower hospitalization rates among patients with behavioural adherence contracts than among controls [17].

The purpose of this paper is to describe the aims, design, and methods of TAKE-IT, a multicentre, prospective, parallel arm, open-label, randomized controlled trial funded by the American National Institutes of Health, National Institutes of Diabetes, Digestive and Kidney diseases (NIDDK; R01DK092977). The study began in Sept. 2011 and will run until June 2016.

## Study aims

The primary aim of TAKE-IT is to characterize and compare, over the 12-month intervention interval, adherence to immunosuppressive medications in the intervention and control groups.

**Figure 1:**
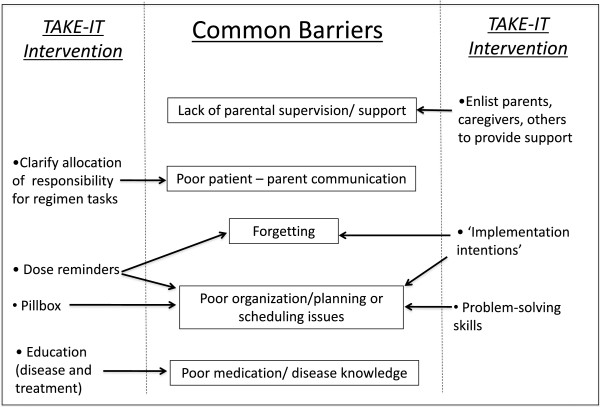
Common barriers to medication adherence are indicated, along with the components of the TAKE-IT intervention that address each.

The TAKE-IT intervention includes **educational**, **organizational**, and **behavioural** components to target common adherence barriers (Figure 1), and incorporates many of the adherence-promoting strategies previously identified as helpful in prior trials, and by families of adolescents with kidney transplants [18]. We hypothesize that, compared with the control group, the intervention group will have significantly better adherence to immunosuppressive medication, which is maintained over the study interval.

A secondary aim of TAKE-IT is to compare the intervention and control groups with respect to graft outcomes. Another secondary aim is to identify independent associations between adherence and healthcare system factors, including characteristics of the treating team, insurance status, Canadian vs. American system, and accessibility to care.

## Methods/Design

### Study organization

**Figure 2:**
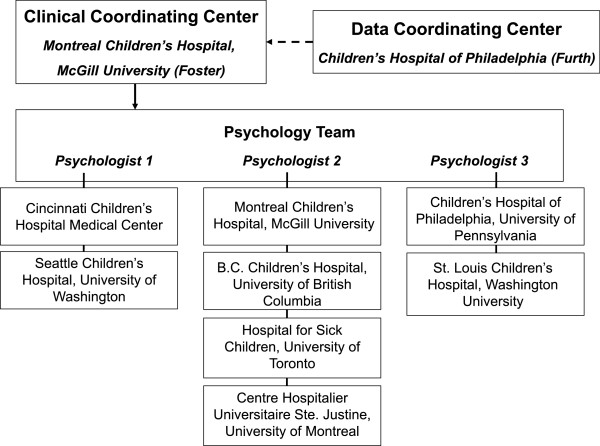
This figure shows the organizational structure of the TAKE-IT study.

Figure 2 shows the organizational structure of the 8 kidney transplant centers across Canada and the United States participating in TAKE-IT. TAKE-IT adheres to the Declaration of Helsinki, and was approved by the Research Ethics Boards of all sites (McGill University Health Centre Research Ethics Board, The Hospital for Sick Children Research Ethics Board, University of British Columbia Children’s and Women’s Health Centre Research Ethics Board, The Children’s Hospital of Philadelphia Institutional Review Board, The Washington University in St. Louis Human Research Protection Office, The Seattle Children’s Hospital Institutional Review Board, The Cincinnati Children’s Hospital Medical Center Institutional Review Board, and the Centre Hospitalier Universitaire Ste-Justine Research Ethics Board). Informed consent is obtained from all participants and/or parents.

### Study population

TAKE-IT will enrol 176 kidney transplant recipients between 11 and 24 years old, who are at least 3 mo. post-transplant, and have a functioning graft. Patients with neurocognitive disabilities severe enough to preclude participation in study procedures, those taking immunuosuppressive medications exclusively in liquid formulation, and those unable to communicate comfortably in English (or French – Montreal sites only) will be excluded.

### Recruitment

Participants will be recruited from the pediatric kidney transplant clinics at participating centers and their affiliated adult clinics over a 30-month interval. The recruitment goal of 176 participants accounts for up to 15% drop out, with the aim of having at least 150 participants complete the study.

### Baseline assessment

Participants are evaluated at a baseline visit timed to coincide with a regularly scheduled clinic visit. At the baseline visit, detailed demographic information (sex, race, education of participant and parents, family structure, household income, etc.) is collected, in addition to information on medical history, such as primary kidney disease, type and duration of renal replacement therapy prior to transplant, donor source, number of prior transplants, current level of graft function, and co-morbid conditions. Detailed information is also collected on immunosuppressive medications, including dosing times, and on concomitant medications, as well as on accessibility to care.

**Figure 3:**
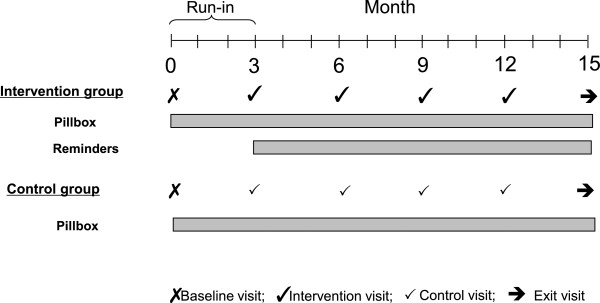
**The timeline of study procedures is indicated, including the schedule of intervention and control visits.** The first 3 months of the study constitutes a run-in period during which patients are not yet randomized to the intervention or control group, and no intervention is applied.

Participants and their parents complete the validated adolescent and parent versions of the Medication Barriers Survey (AMBS and PMBS) [19], the Allocation of Treatment Responsibility (ATR) survey [20], and the Medical Adherence Measure Medication Module (MAM-MM) [21]. All questionnaires were translated into French by a professional translator and underwent basic linguistic validation [22].All participants are given an electronic pillbox for adherence monitoring at the baseline visit and taught how to use it. The dose reminder functions of the pillboxes are disabled for all participants for the first 3 months of the study, which constitutes a run-in period. No intervention is applied during the run-in. Figure 3 provides a timeline of study procedures.

All participants receive usual care from their clinical treating team. Clinical care providers are free to continue to use their usual methods of assessing adherence, and to use their usual methods helping patients to improve adherence. However, no information collected for study purposes is shared with the treating team. The study protocol does not dictate the level of parental involvement in medication administration.

### Randomization and blinding

Participants are randomized at enrolment in age strata (11–13 y., 14–16 y., 17–19 y., 20–24), by site, in blocks of 4. Allocation to intervention or control group is concealed from participants, clinical care providers, and study personnel at enrolment and during the first two months of the run-in period. Group allocation is revealed to study personnel one month prior to the 3-month visit. Blinding is not feasible to maintain with the proposed multi-component intervention.

### Adherence monitoring

There is no perfect method of measuring adherence; in the absence of a true gold standard, electronic monitoring is considered the best available method [23]. In the TAKE-IT study, immunosuppressive medication adherence is monitored using an electronic multi-dose pillbox that is coupled to a personalized, password-protected, medication management website. The pillbox is managed by study personnel; participants do not have access to the website. When an individual compartment of the pillbox is opened, a signal is sent to the company, and a date and time ‘stamp’ is recorded in the electronic record of the patient to whom the device is registered. The pillbox requires a power supply, but has a back-up battery with about 8 hours of life.

Because the electronic pillboxes cannot be carried during daily activities, participants who take medication doses away from home must remove the medication from the device prior to leaving home; participants are asked to either keep a log of medication dosing times when not at home or to send the study staff a text message when they take a dose away from the pillbox [7].

### Intervention

The TAKE-IT intervention simultaneously targets several of the most common barriers to adherence in adolescents, and can be administered as a component of routine clinic visits through relatively brief interactions between a member of the study team, called the ‘Coach’ , and the patient and family.

Participants randomized to the intervention arm receive education on immunosuppressive medications, and along with one or both parents and the trained site Coach, form an Adherence Support Team (AST). The Coach guides the AST to clarify the responsibility of each AST member for medication adherence [20], and uses a novel approach – ‘**Action-Focused Problem-Solving**’ – to address personal barriers to adherence identified as important to the participant using the validated AMBS/PMBS [19]. Action-focused problem-solving incorporates two complementary and well-established behavioural approaches: *problem-solving* and *implementation intentions*.

Problem-solving skills training enables individuals to elicit a variety of potentially effective solutions to a problem and increases the probability that the most effective solution will be chosen [24,25]. Better problem-solving skills have been associated with improved adherence across a number of pediatric populations [14,26,27]. Adherence interventions aimed at increasing problem-solving skills have enhanced adherence to life-long treatment regimens in diabetes, HIV, and asthma [28-30].

Implementation intentions are concrete action plans in which an individual specifies, in an if-then contingency format, when, where and how he or she will perform a behaviour, with the goal of developing habits that promote adherence [31,32]. Forming implementation intentions has been shown to make the execution of a plan automatic – to create a habit [33,34]. Applied to medication adherence, implementation intentions increase the likelihood that a medication is persistently taken on time. Implementation intentions increased medication adherence by 18% in a randomized controlled trial of adults with epilepsy [31]. Intervention arm participants may also choose to receive text message, email, or visual cue dose reminders throughout the study. The initial intervention visit (~2 hr.) is boosted with briefer (20–30 min.) sessions at 3-month intervals during which participants review and update the implementation intentions. At follow-up visits, the electronic adherence monitoring data from the prior 3 months is reviewed with participants in the intervention arm in order to help identify days of the week and times of day that are most problematic, and to guide the development and revision of implementation intentions. Feedback of adherence data was identified as one of the most successful methods of improving adherence in a systematic review of adherence trials [35].

### Control condition

Control participants use the electronic pillbox, and have study visits with the Coach at the same intervals as intervention participants. During these visits, the participants are given the opportunity to talk with the Coach in general terms about their treatment and how they feel it is going, but adherence is not specifically discussed.

### Training and monitoring

Coaches do not require a specific professional background. However, all Coaches undergo an intensive 2-day, in-person training session led by a study psychologist. These training sessions focus on active listening skills and non-judgmental interaction with patients, and teach coaches to administer the intervention and control sessions through didactic sessions and a series of role-plays. Face-to-face training is followed up with one-on-one phone meetings with a study psychologist to review areas noted to be in need of improvement and to make specific plans for additional training if needed. In order to monitor treatment integrity and coach competency throughout the study, all of the intervention and control group sessions are audio-recorded. A supervising psychologist reviews audio-recordings of the first two administrations of each intervention session by each coach for delivery competency. Twenty-five percent of the recorded sessions from each site will be randomly selected and evaluated by a research assistant who is blind to patient identity but not group assignment for intervention fidelity and for competency of intervention delivery by the study psychologists. If deficiencies are identified either with treatment fidelity or competency, these are addressed directly with the Coach by the supervising psychologist. Coaches receive monthly supervision with a licensed clinical psychologist to maintain proficiency levels throughout the course of the project.

### Follow-up and retention

All participants are followed up in person, in conjunction with regular clinic visits, at 3-month intervals over a 15-month period. Study personnel do not share any information collected for study purposes with the treating team. Participants receive modest financial compensation for each study visit attended, as well as token incentive payments for use of the electronic pillbox (determined based on evidence that the box is turned on and communicating with the server, and being refilled regularly). Participants also receive study newsletters, birthday, and holiday cards.

### Study outcomes

Table 1 indicates the **adherence**, **graft**, and **adverse** outcomes being measured in all participants. The planned primary outcome is daily "*taking adherence*", defined as the proportion of daily prescribed doses taken, as measured using electronic monitoring (EM). Because no one method of measuring adherence is considered a gold standard, we will measure adherence in several ways including pharmacy dispensing records, self-report using the MAM-MM, and variability in tacrolimus or sirolimus trough levels [36-38]. We will also consider a composite of variability in tacrolimus or sirolimus trough levels and self-report such as the ‘system for integrated adherence monitoring’ [39].

**Table 1:** Outcome measures

**Category**	**Measures**	**Frequency**
**Adherence outcomes**	**Electronic monitoring**	Continuously monitored
	● Taking adherence (% prescribed doses taken each day)	
	● Timing adherence (% doses taken within 2 hours of scheduled time each day)	
	● Drug holidays (missing ≥2 consecutive doses)	
	**Pharmacy refills**	All refills during study interval
	● Taking adherence (% prescribed doses taken over entire intervention period)	
	**Variability in tacrolimus or sirolimus trough levels**	Monthly levels as measured for clinical care
	● Standard deviation calculated over 6-month intervals	
	**Self-report** (Medical Adherence Measure- Medication Module)	3-month intervals
	● Taking adherence (% prescribed doses taken, in 3-month intervals)	
	● Timing adherence (% doses taken within 2 hours of scheduled time, in 3-month intervals)	
	● Drug holidays (missing ≥2 consecutive doses)	
**Graft outcomes**	**Graft failures or deaths** (# failures/person-year of follow-up)	All failures during study interval
	● Failure defined as loss of graft function requiring return to dialysis or death from any cause	
	**Acute rejections** (# rejections/person-year of follow-up)	All rejections during study interval
	● Biopsy-proven and presumed rejections, defined as rejections diagnosed by the treating physician based on >20% rise in creatinine	
	Percent change in estimated glomerular filtration rate (eGFR)*	
	● Calculated as:	
	(eGFR at start of intervention – eGFR at study exit) ÷ eGFR at start of intervention	
**Adverse outcomes**	Deaths	All deaths during study interval
	Opportunistic viral infections (CMV, EBV, biopsy-proven polyoma virus nephropathy)	All infections during study interval
	Hospitalizations (# hospitalizations/ person-year of follow-up)	All hospitalizations during study interval
	Other medical conditions requiring treatment (# conditions/ person-year of follow-up)	All conditions during study interval

*eGFR is calculated from height and IDMS-standardized serum creatinine using the updated Schwartz formula [40,41].

### Additional data

#### Treating center and healthcare system characteristics

Information [42] regarding practice patterns and characteristics of the healthcare organization will be collected from each of the sites. Examples include number of kidney transplant patients followed at the center, whether a dedicated pharmacist or psychologist regularly interacts with patients as a part of the transplant team, the patient: full-time transplant nurse ratio, methods that patients may use to communicate with the treating team (phone/email/text message), and the frequency of clinic visits and blood monitoring for stable patients. Additional factors [42], indicating characteristics of the healthcare system within which the patient is cared for (adult vs. pediatric, Canadian vs. U.S.), healthcare insurer, medication insurer, and average monthly out-of-pocket expenses will also be recorded.

#### Accessibility to care

The following information will be collected at enrolment: distance of residence from treating center, and access to phone/email/text message support from the treating team. At enrolment, and subsequently every 3 months, participants will be asked if they needed any of the following but could either not afford to get them, or the services were unavailable: prescription medications, medical visits with the transplant team or with a primary care provider [43,44].

### Statistical analysis plan

The primary analysis will use intention-to-treat principles; as-treated secondary analyses will also be done. Daily taking adherence will be determined for each participant for each day of follow-up [45]; the median daily taking adherence will be determined for participants in each of the intervention and control groups, and plotted against time. This will allow assessment of changes in adherence over the intervention interval for each group. We will calculate the area under the curve (AUC) for the entire intervention interval for each group, and compare AUCs using a two-sided, independent two samples t-test or Wilcoxon test. Greater AUC will reflect greater adherence. To account for possible imbalances between groups, and to test for differences in the patterns of change in taking adherence over time between the intervention and control groups, we will use linear mixed-effects models, adjusting for potential confounders. We will use the same approach to analyze the secondary adherence outcomes.

Percent change in estimated glomerular filration rate (eGFR) over the intervention interval will be compared between groups using a 2-sided, independent two samples t-test or a Wilcoxon rank sum test, as appropriate to the distribution of the data. Acute rejections and graft failures will each be expressed as a rate per person-month of observation, and compared between intervention and control groups using Poisson regression.

The multivariable linear mixed-effects models developed for the primary aim will be extended to include center and system characteristics and accessibility factors that are fixed over time (i.e. Canada vs. US), as well as time-varying center, system, and accessibility variables (i.e. insurance status, perceived accessibility). Similar analyses will be undertaken considering secondary adherence outcomes. We will use these models to identify center, system, and accessibility characteristics that are independently associated with adherence.

### Power considerations

Our preliminary electronic monitoring studies in adolescent transplant recipients found a mean (±standard deviation (SD)) taking adherence of 79% ± 33 at one site and 78% ± 29 at another site (unpublished). With 75 participants per group, setting alpha at 0.05, we will have 85% power to detect a 20% difference in taking adherence, using a two group t-test, assuming a common SD of 32 (40% of the mean). Preliminary studies suggested substantially lower variability in adherence after intervention compared with before. If the SD is 25% in the intervention group, and 40% in the control group, we will have 95% power to detect a 20% difference in taking adherence, or 80% power to detect a 16% difference. Based on preliminary studies indicating a 26% improvement in adherence after intervention with dose reminders, and on published work [12], it is reasonable to expect a 20% improvement in adherence when a single level intervention is applied. Effect sizes may be larger with a multi-level intervention.

Due to low event rates and large variability in graft function, statistical power is extremely limited to detect differences between intervention and control groups for graft outcomes; these analyses are considered exploratory.

### Progress

**Figure 4:**
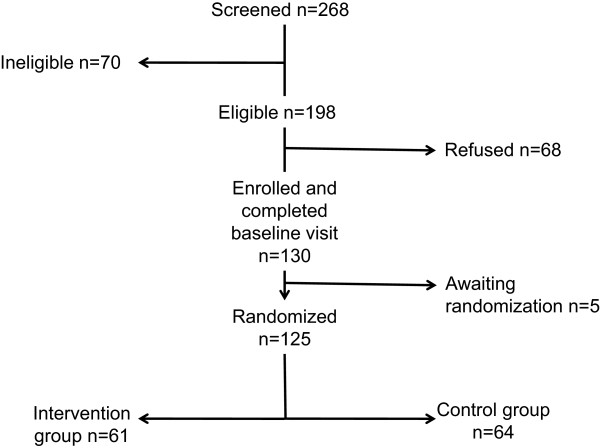
**As of March 31, 2014, 268 patients in the eligible age range had been screened for participation.** Of these, 70 met exclusion criteria (including significant neurocognitive disability, inability to communicate in English or French, imminent graft failure, imminent transfer to adult care). Of the 198 who were eligible to participate, 130 agreed (66%) and had completed a baseline visit. Randomization occurs 1 month before the 3-month visit; 125 participants had been randomized as of March 31, 2014.

Recruitment began in February 2012. Figure 4 shows the numbers of patients in the eligible age range who were screened, enrolled, and randomized to date. As of March 31, 2014, 130 of the 198 adolescents who were approached had agreed to participate (66%). Table 2 shows the demographic, medical, and treatment characteristics, by group, of the 125 participants with known randomization allocation to date. Reasons for refusal fall into 3 general categories: 25% refused to meet with study personnel to hear more about the study; 50% felt that they had no problem with adherence and therefore had no reason to participate; 25% felt it would take too much time or would add expense.

**Table 2:** Baseline demographic and disease/treatment characteristics of participants randomized to date

	**Control**	**Intervention**
n	64	61
Male (%)	39 (60.9)	34 (55.7)
Median age (IQR)	15.9 (13.6 – 17.5)	16.2 (14.0 – 17.8)
Race (%)		
White	42 (65.6)	43 (70.5)
Black	9 (14.1)	6 (9.8)
Asian	4 (6.3)	3 (4.9)
American Indian/Alaska Native	2 (3.1)	1 (1.7)
Hawaiian/Other Pacific Islander	2 (3.1)	0 (0)
Other	5 (7.8)	8 (13.1)
Hispanic (%)	4 (6.3)	7 (11.5)
Median years since transplant (IQR)	3.7 (0.8-7.5)	4.4 (1.5-8.4)
Primary disease (%)		
CAKUT	30 (46.9)	21 (34.4)
Glomerulonephritis	3 (4.7)	6 (9.8)
FSGS	6 (9.4)	9 (14.8)
Other	23 (35.9)	21 (34.4)
Missing	2 (3.1)	4 (6.6)
Number of immunosuppressive medications (%)		
1	3 (4.7)	4 (6.6)
2	27 (42.2)	21 (34.4)
3	34 (53.1)	35 (57.4)
4	0 (0.0)	1 (1.6)
Median total number of all medications (IQR)	6 (5 - 9)	7 (5 - 9)

*CAKUT* Congenital anomalies of the kidneys and urinary tract, *FSGS* Focal and segmental glomerulosclerosis.

## Discussion

A 2008 U.S. National Institutes of Health consensus conference on immunosuppressive medication non-adherence highlighted the consequences of and risk factors for non-adherence, and made an urgent call for adequately powered randomized trials to test interventions to improve adherence in the high-risk adolescent transplant population [8]. TAKE-IT was designed to address the need for effective interventions to improve medication adherence in youth with kidney transplants.

An important feature of the TAKE-IT intervention is that it is administered repeatedly at regular intervals – an intervention approach [46,47] demonstrated to offer better sustained treatment effects than interventions delivered in single session or concentrated formats [48,49]. Repeated intervention sessions provide an opportunity to address not only the barriers present at initiation of the intervention, but can pre-empt new barriers to prevent future non-adherence, as barriers change over time [7,21]. A similar ‘continuous self-improvement’ adherence intervention approach in adult kidney transplant recipients, involving regular contact with participants, showed promise [50]. Furthermore, the TAKE-IT intervention is clinic-based, and integrated with usual clinical care, making it feasible for application in practice. It may be possible to include lay coaches, supported by a psychologist, into the clinical care team to support adherence. The feasibility of widespread use of EM devices in clinical practice will require further study.

In addition to determining the effectiveness of the intervention, this study will provide important information on the time and resources needed to apply such an intervention as a part of clinical care. It will not be possible for us to determine which component(s) of the TAKE-IT intervention are most powerful in promoting adherence. However, if effective, this will be the first intervention demonstrated to improve adherence in a randomized trial for adolescent kidney transplant recipients, and as such will represent a major advance in the standard of care. Future studies will refine intervention strategies.

## Abbreviations

TAKE-IT: Teen Adherence in Kidney Transplantation Effectiveness of Intervention Trial; AMBS: Adolescent version of the Medication Barriers Survey; PMBS: Parent version of the Medication Barriers Survey; ATR: Allocation of Treatment Responsibility; MAM-MM: Medical Adherence Measure Medication Module; AST: Adherence Support Team; AUC: Area under the curve; eGFR: Estimated glomerular filration rate.

## Competing interests

The authors declare that they have no competing interests.

## Authors’ contributions

BJF is the study Principle Investigator and held primary responsibility for study design. She directs conduct of the study and drafted the manuscript. AP is the lead study Psychologist and participated in study design and development of the intervention. She is responsible for coach training, and supervises the work of study psychologists. She reviewed and edited the manuscript. HZ is the study biostatistician responsible for randomization, data management, and data analyses. He analyzed the data needed to describe progress to date, and reviewed and edited the manuscript. SF is the study co-Principle Investigator and holds primary responsibility for directing data management. She reviewed and edited the manuscript. All authors read and approved the final manuscript.

## Pre-publication history

The pre-publication history for this paper can be accessed here:

http://www.biomedcentral.com/1471-2369/15/139/prepub
